# 1-{(1*Z*)-1-[3-(2,4-Dichloro­phen­oxy)prop­oxy]-1-(2,4-difluoro­phen­yl)prop-1-en-2-yl}-1*H*-1,2,4-triazole

**DOI:** 10.1107/S1600536812017874

**Published:** 2012-05-19

**Authors:** Yuan-yuan Luan, Yong-hong Hu, Song Guo, Jing Zhu, Wen-ge Yang

**Affiliations:** a College of Pharmaceutical Science, Nanjing University of Technology, Xinmofan Road No.5 Nanjing, Nanjing 210009, People’s Republic of China; b Jiangsu Engineering Technology Research Center of Polypeptide Pharmaceuticals, College of Life Science and Pharmaceutical Engineering, Nanjing University of Technology, Xinmofan Road No.5 Nanjing, Nanjing 210009, People’s Republic of China

## Abstract

In the title compound, C_20_H_17_Cl_2_F_2_N_3_O_2_, the triazole ring makes dihedral angles of 28.0 (3) and 72.5 (2)° with the 2,4-dichloro­pheny and 2,4-difluoro­phenyl rings, respectively, and the mol­ecule adopts a *Z*-conformation about the C=C double bond. In the crystal, C—H⋯O and C—H⋯N hydrogen bonds link the mol­ecules.

## Related literature

For a related structure and background to triazoles and further synthetic details, see: Shen *et al.* (2012 ▶).
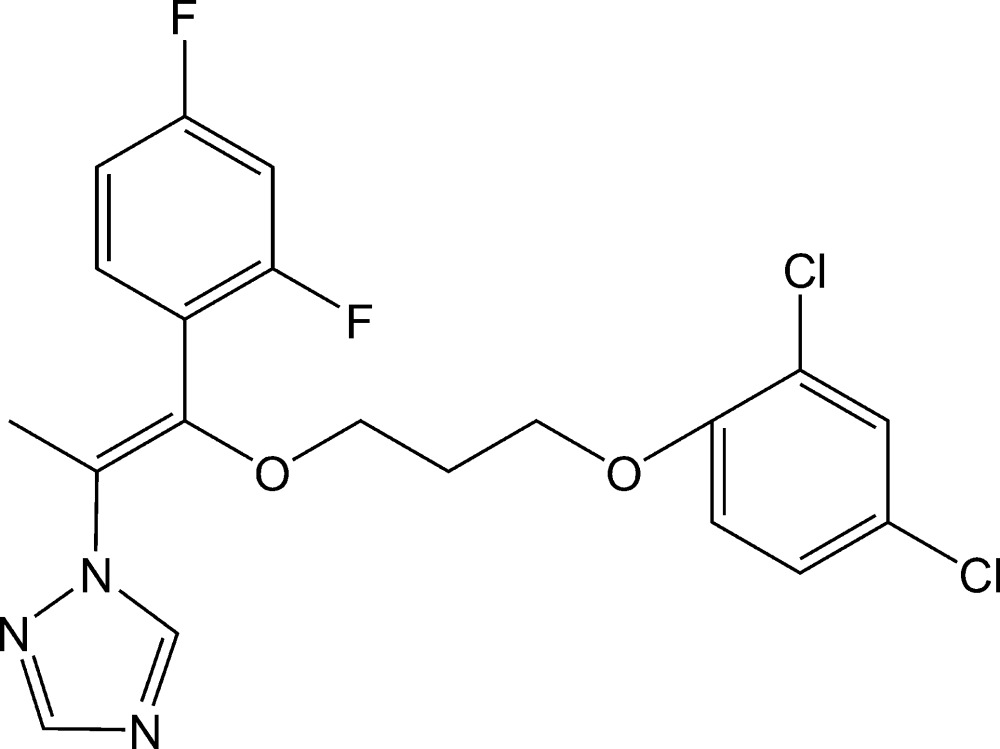


## Experimental

### 

#### Crystal data

C_20_H_17_Cl_2_F_2_N_3_O_2_
*M* *_r_* = 440.27
Triclinic, 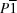

*a* = 7.4380 (15) Å
*b* = 8.7600 (18) Å
*c* = 15.892 (3) Å
α = 89.48 (3)°
β = 84.57 (3)°
γ = 73.74 (3)°
*V* = 989.4 (3) Å^3^
*Z* = 2
Mo *K*α radiation
μ = 0.37 mm^−1^
*T* = 293 K
0.30 × 0.20 × 0.10 mm

#### Data collection

Enraf–Nonius CAD-4 diffractometer
Absorption correction: ψ scan (North *et al.*, 1968 ▶) *T*_min_ = 0.898, *T*_max_ = 0.964
3933 measured reflections
3628 independent reflections
2564 reflections with *I* > 2σ(*I*)
*R*_int_ = 0.022
3 standard reflections every 200 reflections intensity decay: 1%

#### Refinement

*R*[*F* ^2^ > 2σ(*F* ^2^)] = 0.054
*wR*(*F* ^2^) = 0.158
*S* = 1.01
3628 reflections
262 parameters
2 restraints
H-atom parameters constrained
Δρ_max_ = 1.21 e Å^−3^
Δρ_min_ = −0.32 e Å^−3^

### 

Data collection: *CAD-4 EXPRESS* (Enraf–Nonius, 1994 ▶); cell refinement: *CAD-4 EXPRESS*; data reduction: *XCAD4* (Harms & Wocadlo, 1995 ▶); program(s) used to solve structure: *SHELXS97* (Sheldrick, 2008 ▶); program(s) used to refine structure: *SHELXL97* (Sheldrick, 2008 ▶); molecular graphics: *SHELXTL* (Sheldrick, 2008 ▶); software used to prepare material for publication: *PLATON* (Spek, 2009 ▶).

## Supplementary Material

Crystal structure: contains datablock(s) global, I. DOI: 10.1107/S1600536812017874/hb6698sup1.cif

Structure factors: contains datablock(s) I. DOI: 10.1107/S1600536812017874/hb6698Isup2.hkl

Supplementary material file. DOI: 10.1107/S1600536812017874/hb6698Isup3.cml

Additional supplementary materials:  crystallographic information; 3D view; checkCIF report

## Figures and Tables

**Table 1:** Hydrogen-bond geometry (Å, °)

*D*—H⋯*A*	*D*—H	H⋯*A*	*D*⋯*A*	*D*—H⋯*A*
C4—H4*A*⋯O1^i^	0.93	2.52	3.421 (4)	163
C17—H17*A*⋯N2^ii^	0.93	2.56	3.400 (4)	151

Symmetry codes: (i) 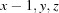; (ii) 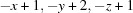.

**Fig. 1.**
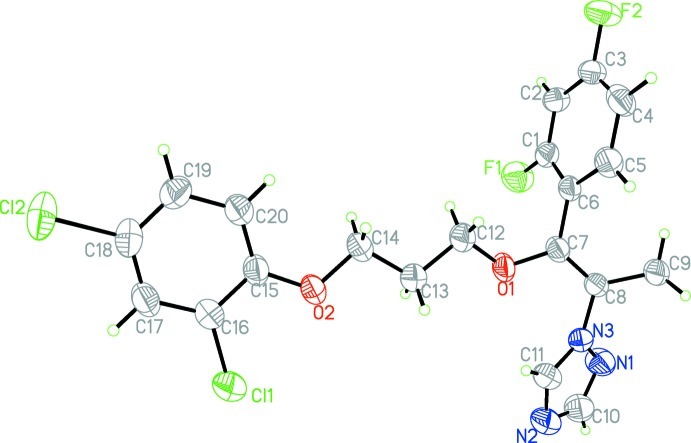
The molecular structure of the title molecule, with displacement ellipsoids drawn at 30% probability levels.

**Fig. 2.**
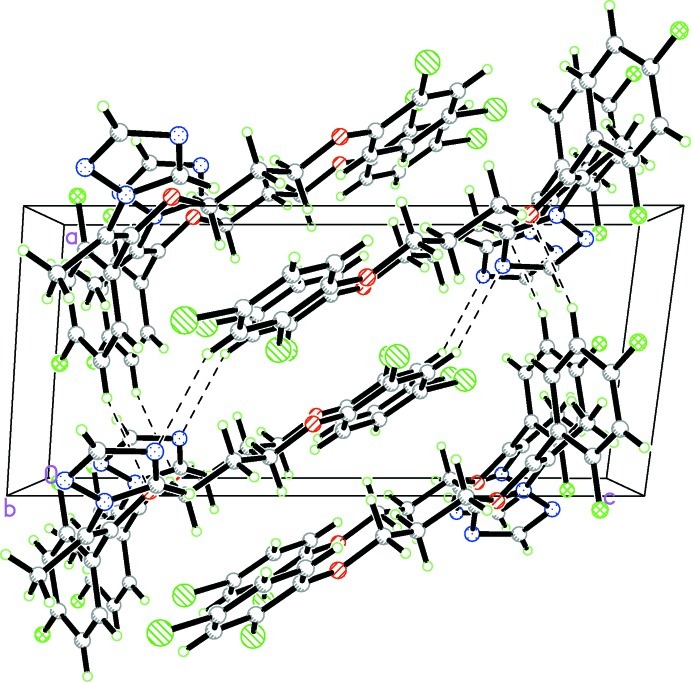
The packing diagram of the title compound. Hydron bonds are shown as dashed lines.

## supplementary crystallographic information

### Comment

As part of our studies on the synthesis of new triazole derivatives (Shen *et al.* 2012), the crystal structure of the title compound was determined.

In the molecular structure of the title compound the double bond is Z configurated. In the crystal, C-H···O and C-H···N hydrogen bonds link the molecules, in which they seem to be effective in the stabilization of the structure. (Table 1 and Fig. 2).

### Experimental

3 g (0.01 mol) 1-(2,4-difluorophenyl)-2-(1,2,4-triazol)-1-y1)propan-1-one, 10 g of a 50% aqueous sodium hydroxide, 15 ml toluene and 1.5 ml of a 40% aqueous solution of tetrabutyl ammonium hydroxide are mixed and heated to 323.15 K under vigorous stirring. 2.8g (0.01 mol) 1-bromo-3-(2.4-dichlorophenoxy)-propane, dissolved in 10 ml toluene, is instilled into the stirred and warmed solution in the course of 10 h. The mixture is subsequently stirred for another 20 h at 323.15 K. The reaction mixture is mixed with as much water and chloroform so that the aqueous phase becomes lighter than the organic phase. Thereafter, the organic and aqueous phases are separated. The organic phase is dried with sodium sulfate. The solvents are distilled under reduced pressure. The impure product herein is subsequently crystallized from a 1:1 mixture of ethyl acetate and ethanol. The purified product may be analytically identified as an approximately pure *Z*-isomer. Colourless blocks of the title compound were obtained by slow evaporation of an ethanol solution.

### Refinement

H atoms were positioned geometrically with C—H = 0.93 and 0.97 Å for aromatic and methylene H atoms, respectively, constrained to ride on their parent atoms, with *U*_iso_(H) = 1.2 (or 1.5 for methyl groups) times *U*_eq_(C).

### Crystal data

**Table:**

C_20_H_17_Cl_2_F_2_N_3_O_2_	*Z* = 2
*M**_r_* = 440.27	*F*(000) = 452
Triclinic, *P*1	*D*_x_ = 1.478 Mg m^−^^3^
Hall symbol: -P 1	Mo *K*α radiation, λ = 0.71073 Å
*a* = 7.4380 (15) Å	Cell parameters from 25 reflections
*b* = 8.7600 (18) Å	θ = 9–13°
*c* = 15.892 (3) Å	µ = 0.37 mm^−^^1^
α = 89.48 (3)°	*T* = 293 K
β = 84.57 (3)°	Block, colorness
γ = 73.74 (3)°	0.30 × 0.20 × 0.10 mm
*V* = 989.4 (3) Å^3^	

### Data collection

**Table:**

Enraf–Nonius CAD-4 diffractometer	2564 reflections with *I* > 2σ(*I*)
Radiation source: fine-focus sealed tube	*R*_int_ = 0.022
Graphite monochromator	θ_max_ = 25.4°, θ_min_ = 1.3°
ω/2θ scans	*h* = 0→8
Absorption correction: ψ scan '(North *et al.*, 1968)	*k* = −10→10
*T*_min_ = 0.898, *T*_max_ = 0.964	*l* = −19→19
3933 measured reflections	3 standard reflections every 200 reflections
3628 independent reflections	intensity decay: 1%

### Refinement

**Table:**

Refinement on *F*^2^	Primary atom site location: structure-invariant direct methods
Least-squares matrix: full	Secondary atom site location: difference Fourier map
*R*[*F*^2^ > 2σ(*F*^2^)] = 0.054	Hydrogen site location: inferred from neighbouring sites
*wR*(*F*^2^) = 0.158	H-atom parameters constrained
*S* = 1.01	*w* = 1/[σ^2^(*F*_o_^2^) + (0.1*P*)^2^] where *P* = (*F*_o_^2^ + 2*F*_c_^2^)/3
3628 reflections	(Δ/σ)_max_ < 0.001
262 parameters	Δρ_max_ = 1.21 e Å^−^^3^
2 restraints	Δρ_min_ = −0.32 e Å^−^^3^

### Special details

**Table:**

Geometry. All e.s.d.'s (except the e.s.d. in the dihedral angle between two l.s. planes) are estimated using the full covariance matrix. The cell e.s.d.'s are taken into account individually in the estimation of e.s.d.'s in distances, angles and torsion angles; correlations between e.s.d.'s in cell parameters are only used when they are defined by crystal symmetry. An approximate (isotropic) treatment of cell e.s.d.'s is used for estimating e.s.d.'s involving l.s. planes.
Refinement. Refinement of *F*^2^ against ALL reflections. The weighted *R*-factor *wR* and goodness of fit *S* are based on *F*^2^, conventional *R*-factors *R* are based on *F*, with *F* set to zero for negative *F*^2^. The threshold expression of *F*^2^ > σ(*F*^2^) is used only for calculating *R*-factors(gt) *etc*. and is not relevant to the choice of reflections for refinement. *R*-factors based on *F*^2^ are statistically about twice as large as those based on *F*, and *R*- factors based on ALL data will be even larger.

### Fractional atomic coordinates and isotropic or equivalent isotropic displacement parameters (Å^2^)

**Table:**

	*x*	*y*	*z*	*U*_iso_*/*U*_eq_	
Cl1	0.48843 (15)	0.85730 (11)	0.59207 (6)	0.0793 (3)	
O1	0.0231 (3)	1.0877 (2)	0.22722 (12)	0.0563 (6)	
F1	0.0405 (2)	0.8859 (2)	0.07385 (13)	0.0733 (5)	
N1	0.0846 (4)	1.4711 (3)	0.11743 (16)	0.0601 (7)	
C1	−0.1446 (4)	0.9223 (3)	0.09377 (16)	0.0430 (6)	
Cl2	0.39808 (13)	0.30882 (11)	0.71824 (5)	0.0769 (3)	
F2	−0.5362 (3)	0.7795 (2)	0.05601 (13)	0.0762 (6)	
O2	0.2476 (3)	0.8160 (2)	0.46936 (12)	0.0567 (5)	
C2	−0.2436 (4)	0.8278 (3)	0.06304 (18)	0.0494 (7)	
H2A	−0.1844	0.7386	0.0291	0.059*	
N2	0.1804 (4)	1.4856 (3)	0.24639 (18)	0.0661 (7)	
N3	0.0067 (3)	1.3826 (2)	0.17311 (13)	0.0412 (5)	
C3	−0.4343 (4)	0.8715 (3)	0.08482 (18)	0.0502 (7)	
C4	−0.5252 (4)	1.0021 (4)	0.13428 (18)	0.0536 (7)	
H4A	−0.6547	1.0283	0.1479	0.064*	
C5	−0.4209 (4)	1.0939 (3)	0.16344 (18)	0.0491 (7)	
H5A	−0.4813	1.1835	0.1969	0.059*	
C6	−0.2260 (3)	1.0556 (3)	0.14398 (15)	0.0391 (6)	
C7	−0.1090 (4)	1.1539 (3)	0.17330 (15)	0.0403 (6)	
C8	−0.1167 (3)	1.2983 (3)	0.14444 (15)	0.0388 (6)	
C9	−0.2440 (4)	1.3857 (3)	0.08190 (18)	0.0517 (7)	
H9A	−0.3213	1.3218	0.0657	0.078*	
H9B	−0.1700	1.4075	0.0329	0.078*	
H9C	−0.3226	1.4840	0.1069	0.078*	
C10	0.1855 (4)	1.5294 (4)	0.1652 (2)	0.0635 (8)	
H10A	0.2557	1.5965	0.1441	0.076*	
C11	0.0658 (4)	1.3936 (3)	0.24882 (19)	0.0541 (7)	
H11A	0.0312	1.3434	0.2970	0.065*	
C12	0.0011 (4)	0.9622 (3)	0.28022 (18)	0.0532 (7)	
H12A	0.0406	0.8621	0.2488	0.064*	
H12B	−0.1297	0.9811	0.3020	0.064*	
C13	0.1210 (4)	0.9566 (3)	0.35150 (17)	0.0516 (7)	
H13A	0.2475	0.9540	0.3288	0.062*	
H13B	0.0706	1.0520	0.3865	0.062*	
C14	0.1277 (4)	0.8136 (4)	0.40447 (18)	0.0542 (7)	
H14A	0.0024	0.8167	0.4293	0.065*	
H14B	0.1770	0.7173	0.3701	0.065*	
C15	0.2795 (4)	0.6921 (3)	0.52364 (16)	0.0463 (6)	
C16	0.3907 (4)	0.7002 (3)	0.58833 (16)	0.0483 (7)	
C17	0.4295 (4)	0.5822 (3)	0.64753 (16)	0.0536 (7)	
H17A	0.5028	0.5890	0.6909	0.064*	
C18	0.3582 (4)	0.4548 (4)	0.64139 (17)	0.0528 (7)	
C19	0.2511 (4)	0.4420 (4)	0.57782 (19)	0.0593 (8)	
H19A	0.2043	0.3545	0.5744	0.071*	
C20	0.2131 (4)	0.5605 (4)	0.51874 (19)	0.0563 (8)	
H20A	0.1418	0.5514	0.4750	0.068*	

### Atomic displacement parameters (Å^2^)

**Table:**

	*U*^11^	*U*^22^	*U*^33^	*U*^12^	*U*^13^	*U*^23^
Cl1	0.1089 (7)	0.0746 (6)	0.0782 (6)	−0.0529 (5)	−0.0461 (5)	0.0141 (4)
O1	0.0545 (12)	0.0545 (11)	0.0728 (13)	−0.0284 (10)	−0.0339 (10)	0.0327 (10)
F1	0.0522 (10)	0.0749 (12)	0.0908 (13)	−0.0154 (9)	−0.0029 (9)	−0.0087 (10)
N1	0.0683 (17)	0.0634 (16)	0.0633 (16)	−0.0394 (14)	−0.0173 (13)	0.0194 (12)
C1	0.0434 (12)	0.0415 (14)	0.0459 (14)	−0.0140 (11)	−0.0079 (11)	0.0103 (11)
Cl2	0.0869 (6)	0.0753 (6)	0.0652 (5)	−0.0163 (5)	−0.0137 (4)	0.0343 (4)
F2	0.0778 (13)	0.0781 (12)	0.0946 (14)	−0.0494 (11)	−0.0350 (11)	0.0096 (10)
O2	0.0700 (13)	0.0610 (12)	0.0528 (11)	−0.0325 (11)	−0.0326 (10)	0.0198 (9)
C2	0.0566 (18)	0.0445 (15)	0.0531 (16)	−0.0213 (13)	−0.0140 (13)	0.0054 (12)
N2	0.0677 (17)	0.0615 (16)	0.0811 (19)	−0.0301 (14)	−0.0314 (14)	0.0039 (14)
N3	0.0425 (12)	0.0370 (11)	0.0479 (12)	−0.0154 (9)	−0.0102 (10)	0.0041 (9)
C3	0.0591 (18)	0.0484 (15)	0.0554 (16)	−0.0294 (14)	−0.0247 (14)	0.0148 (13)
C4	0.0390 (15)	0.0676 (19)	0.0604 (17)	−0.0222 (14)	−0.0149 (13)	0.0189 (15)
C5	0.0455 (16)	0.0507 (15)	0.0533 (16)	−0.0151 (13)	−0.0114 (12)	0.0080 (13)
C6	0.0379 (14)	0.0400 (14)	0.0435 (13)	−0.0149 (11)	−0.0134 (11)	0.0133 (11)
C7	0.0399 (14)	0.0448 (14)	0.0393 (13)	−0.0144 (11)	−0.0117 (11)	0.0072 (11)
C8	0.0400 (14)	0.0402 (13)	0.0404 (13)	−0.0158 (11)	−0.0115 (11)	0.0051 (11)
C9	0.0614 (18)	0.0445 (15)	0.0576 (16)	−0.0222 (13)	−0.0270 (14)	0.0173 (13)
C10	0.064 (2)	0.0578 (18)	0.081 (2)	−0.0335 (16)	−0.0185 (17)	0.0128 (16)
C11	0.0612 (19)	0.0527 (16)	0.0555 (16)	−0.0227 (14)	−0.0211 (14)	0.0025 (13)
C12	0.0614 (18)	0.0523 (16)	0.0539 (16)	−0.0245 (14)	−0.0218 (14)	0.0183 (13)
C13	0.0611 (18)	0.0471 (15)	0.0523 (16)	−0.0202 (14)	−0.0196 (14)	0.0103 (13)
C14	0.0608 (18)	0.0605 (17)	0.0523 (16)	−0.0283 (15)	−0.0276 (14)	0.0165 (14)
C15	0.0486 (16)	0.0548 (16)	0.0406 (14)	−0.0205 (13)	−0.0126 (12)	0.0134 (12)
C16	0.0521 (17)	0.0511 (16)	0.0442 (15)	−0.0157 (13)	−0.0128 (12)	0.0018 (12)
C17	0.0573 (18)	0.0642 (18)	0.0418 (15)	−0.0174 (15)	−0.0177 (13)	0.0059 (13)
C18	0.0496 (16)	0.0602 (18)	0.0456 (15)	−0.0105 (14)	−0.0060 (13)	0.0161 (13)
C19	0.0620 (19)	0.0601 (18)	0.0656 (19)	−0.0311 (15)	−0.0139 (15)	0.0197 (15)
C20	0.0600 (19)	0.0647 (18)	0.0551 (17)	−0.0302 (15)	−0.0230 (14)	0.0168 (14)

### Geometric parameters (Å, º)

**Table:**

Cl1—C16	1.730 (3)	C7—C8	1.329 (3)
O1—C7	1.367 (3)	C8—C9	1.493 (3)
O1—C12	1.416 (3)	C9—H9A	0.9600
F1—C1	1.331 (3)	C9—H9B	0.9600
N1—C10	1.315 (4)	C9—H9C	0.9600
N1—N3	1.365 (3)	C10—H10A	0.9300
C1—C2	1.372 (4)	C11—H11A	0.9300
C1—C6	1.377 (4)	C12—C13	1.499 (4)
Cl2—C18	1.744 (3)	C12—H12A	0.9700
F2—C3	1.359 (3)	C12—H12B	0.9700
O2—C15	1.362 (3)	C13—C14	1.495 (4)
O2—C14	1.430 (3)	C13—H13A	0.9700
C2—C3	1.372 (4)	C13—H13B	0.9700
C2—H2A	0.9300	C14—H14A	0.9700
N2—C11	1.325 (4)	C14—H14B	0.9700
N2—C10	1.343 (4)	C15—C20	1.381 (4)
N3—C11	1.333 (3)	C15—C16	1.394 (4)
N3—C8	1.435 (3)	C16—C17	1.380 (4)
C3—C4	1.366 (4)	C17—C18	1.371 (4)
C4—C5	1.373 (4)	C17—H17A	0.9300
C4—H4A	0.9300	C18—C19	1.368 (4)
C5—C6	1.398 (4)	C19—C20	1.380 (4)
C5—H5A	0.9300	C19—H19A	0.9300
C6—C7	1.490 (3)	C20—H20A	0.9300
			
C7—O1—C12	120.2 (2)	N2—C11—N3	111.0 (3)
C10—N1—N3	102.4 (2)	N2—C11—H11A	124.5
F1—C1—C2	119.0 (3)	N3—C11—H11A	124.5
F1—C1—C6	117.2 (2)	O1—C12—C13	107.3 (2)
C2—C1—C6	123.8 (3)	O1—C12—H12A	110.3
C15—O2—C14	117.3 (2)	C13—C12—H12A	110.3
C1—C2—C3	116.7 (3)	O1—C12—H12B	110.3
C1—C2—H2A	121.7	C13—C12—H12B	110.3
C3—C2—H2A	121.7	H12A—C12—H12B	108.5
C11—N2—C10	102.3 (3)	C14—C13—C12	111.2 (2)
C11—N3—N1	108.8 (2)	C14—C13—H13A	109.4
C11—N3—C8	131.6 (2)	C12—C13—H13A	109.4
N1—N3—C8	119.6 (2)	C14—C13—H13B	109.4
F2—C3—C4	118.9 (3)	C12—C13—H13B	109.4
F2—C3—C2	118.1 (3)	H13A—C13—H13B	108.0
C4—C3—C2	123.0 (3)	O2—C14—C13	107.4 (2)
C3—C4—C5	118.5 (3)	O2—C14—H14A	110.2
C3—C4—H4A	120.8	C13—C14—H14A	110.2
C5—C4—H4A	120.8	O2—C14—H14B	110.2
C4—C5—C6	121.5 (3)	C13—C14—H14B	110.2
C4—C5—H5A	119.3	H14A—C14—H14B	108.5
C6—C5—H5A	119.3	O2—C15—C20	125.4 (2)
C1—C6—C5	116.6 (2)	O2—C15—C16	116.3 (2)
C1—C6—C7	120.4 (2)	C20—C15—C16	118.3 (2)
C5—C6—C7	122.9 (2)	C17—C16—C15	121.0 (3)
C8—C7—O1	118.3 (2)	C17—C16—Cl1	119.6 (2)
C8—C7—C6	123.1 (2)	C15—C16—Cl1	119.3 (2)
O1—C7—C6	118.4 (2)	C18—C17—C16	118.9 (3)
C7—C8—N3	119.7 (2)	C18—C17—H17A	120.5
C7—C8—C9	125.8 (2)	C16—C17—H17A	120.5
N3—C8—C9	114.4 (2)	C19—C18—C17	121.5 (3)
C8—C9—H9A	109.5	C19—C18—Cl2	119.1 (2)
C8—C9—H9B	109.5	C17—C18—Cl2	119.4 (2)
H9A—C9—H9B	109.5	C18—C19—C20	119.3 (3)
C8—C9—H9C	109.5	C18—C19—H19A	120.4
H9A—C9—H9C	109.5	C20—C19—H19A	120.4
H9B—C9—H9C	109.5	C19—C20—C15	121.0 (3)
N1—C10—N2	115.5 (3)	C19—C20—H20A	119.5
N1—C10—H10A	122.2	C15—C20—H20A	119.5
N2—C10—H10A	122.2		
			
F1—C1—C2—C3	−179.4 (2)	C11—N3—C8—C9	141.9 (3)
C6—C1—C2—C3	−0.1 (4)	N1—N3—C8—C9	−38.1 (3)
C10—N1—N3—C11	−0.2 (3)	N3—N1—C10—N2	0.4 (4)
C10—N1—N3—C8	179.8 (2)	C11—N2—C10—N1	−0.4 (4)
C1—C2—C3—F2	−179.4 (2)	C10—N2—C11—N3	0.3 (3)
C1—C2—C3—C4	0.2 (4)	N1—N3—C11—N2	−0.1 (3)
F2—C3—C4—C5	179.7 (2)	C8—N3—C11—N2	179.9 (2)
C2—C3—C4—C5	0.0 (4)	C7—O1—C12—C13	−159.4 (2)
C3—C4—C5—C6	−0.4 (4)	O1—C12—C13—C14	−172.2 (2)
F1—C1—C6—C5	179.1 (2)	C15—O2—C14—C13	−177.7 (2)
C2—C1—C6—C5	−0.2 (4)	C12—C13—C14—O2	178.8 (3)
F1—C1—C6—C7	0.6 (3)	C14—O2—C15—C20	3.4 (4)
C2—C1—C6—C7	−178.7 (2)	C14—O2—C15—C16	−177.6 (3)
C4—C5—C6—C1	0.5 (4)	O2—C15—C16—C17	179.1 (2)
C4—C5—C6—C7	178.9 (2)	C20—C15—C16—C17	−1.8 (4)
C12—O1—C7—C8	159.7 (3)	O2—C15—C16—Cl1	−3.3 (4)
C12—O1—C7—C6	−25.5 (4)	C20—C15—C16—Cl1	175.8 (2)
C1—C6—C7—C8	109.1 (3)	C15—C16—C17—C18	0.7 (4)
C5—C6—C7—C8	−69.4 (4)	Cl1—C16—C17—C18	−177.0 (2)
C1—C6—C7—O1	−65.5 (3)	C16—C17—C18—C19	0.4 (5)
C5—C6—C7—O1	116.1 (3)	C16—C17—C18—Cl2	−177.4 (2)
O1—C7—C8—N3	−2.7 (4)	C17—C18—C19—C20	−0.3 (5)
C6—C7—C8—N3	−177.2 (2)	Cl2—C18—C19—C20	177.5 (2)
O1—C7—C8—C9	176.6 (2)	C18—C19—C20—C15	−0.9 (5)
C6—C7—C8—C9	2.0 (4)	O2—C15—C20—C19	−179.0 (3)
C11—N3—C8—C7	−38.8 (4)	C16—C15—C20—C19	1.9 (5)
N1—N3—C8—C7	141.2 (3)		

### Hydrogen-bond geometry (Å, º)

**Table:**

*D*—H···*A*	*D*—H	H···*A*	*D*···*A*	*D*—H···*A*
C4—H4*A*···O1^i^	0.93	2.52	3.421 (4)	163
C17—H17*A*···N2^ii^	0.93	2.56	3.400 (4)	151

Symmetry codes: (i) *x*−1, *y*, *z*; (ii) −*x*+1, −*y*+2, −*z*+1.

## References

[bb1] Enraf–Nonius (1994). *CAD-4 EXPRESS* Enraf–Nonius, Delft, The Netherlands.

[bb2] Harms, K. & Wocadlo, S. (1995). *XCAD4* University of Marburg, Germany.

[bb3] North, A. C. T., Phillips, D. C. & Mathews, F. S. (1968). *Acta Cryst.* A**24**, 351–359.

[bb4] Sheldrick, G. M. (2008). *Acta Cryst.* A**64**, 112–122. 10.1107/S0108767307043930 18156677

[bb5] Shen, F., Guo, S., Luan, Y.-Y., Wang, K. & Hu, Y.-H. (2012). *Acta Cryst.* E**68**, submited [HB6699]. 10.1107/S1600536812024154 PMC3393281 22807838

[bb6] Spek, A. L. (2009). *Acta Cryst.* D**65**, 148–155. 10.1107/S090744490804362X PMC2631630 19171970

